# Supplementary material for: Electroactive 3D Printed Scaffolds Based on Percolated Composites of Polycaprolactone with Thermally Reduced Graphene Oxide for Antibacterial and Tissue Engineering Applications
Source: Nanomaterials (Basel). 2020 Feb 28;10(3):428. doi: 10.3390/nano10030428 (PMC7152842; doi:10.3390/nano10030428)
Supplement: Supplementary file 1 [file nanomaterials-10-00428-s001.pdf]

Supplementary Section for:

Electroactive 3D Printed Scaffolds based on Percolated Composites of Polycaprolactone with Thermally Reduced Graphene Oxide for Antibacterial and Tissue Engineering Applications

Carolina Angulo-Pineda* [1,2], Kasama Srirussamee [3], Patricia Palma [4], Victor Fuenzalida [5], Sarah H. Cartmell [6] and Humberto Palza*[1,2]

1. Department of Chemical Engineering and Biotechnology and Materials, University of Chile, Santiago 8370456, Chile
2. Millenium Nuclei in Soft Smart Mechanical Metamaterials, Universidad de Chile, Santiago, 8370456, Chile
3. Department of Biomedical Engineering, Faculty of Engineering, King Mongkut's Institute of Technology Ladkrabang (KMITL), Bangkok 10520, Thailand
4. Department of Pathology and Oral Medicine, University of Chile, Santiago 8380492, Chile
5. Department of Physics, University of Chile, Santiago 8370449, Chile
6. Department of Materials, The University of Manchester, Manchester M13 9PL, United Kingdom

* Correspondence: cangulo@u.uchile.cl (C.A.P.); hpalza@ing.uchile.cl (H. P.)

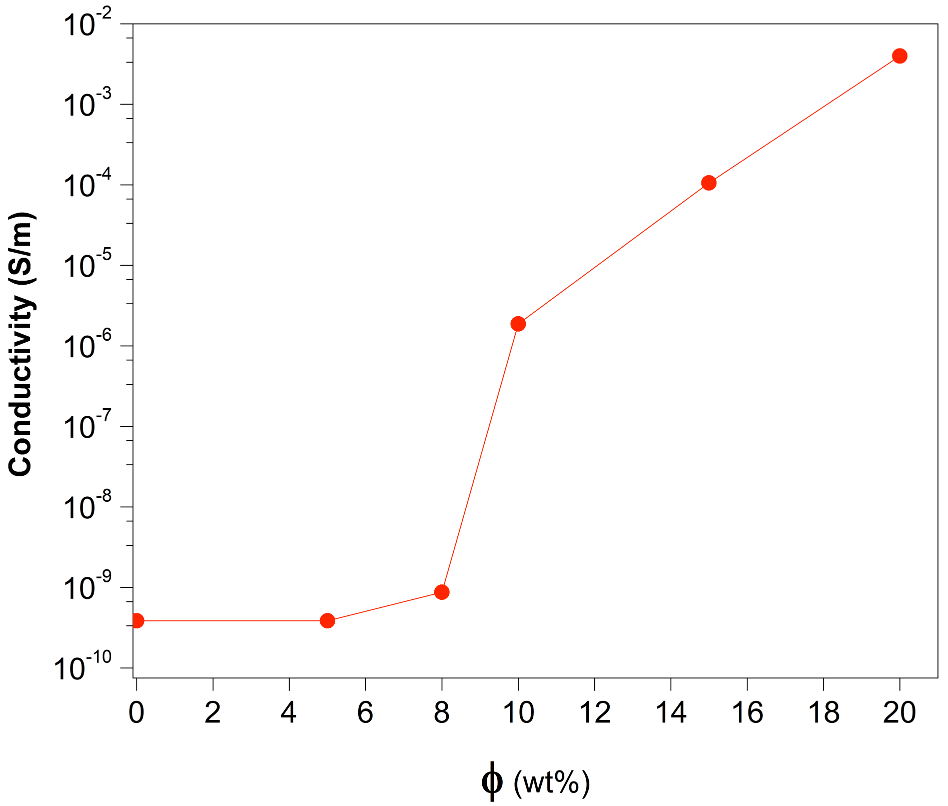

Figure S1: Percolation curve of PCL/TrGO composites at different concentration of conductive particles.

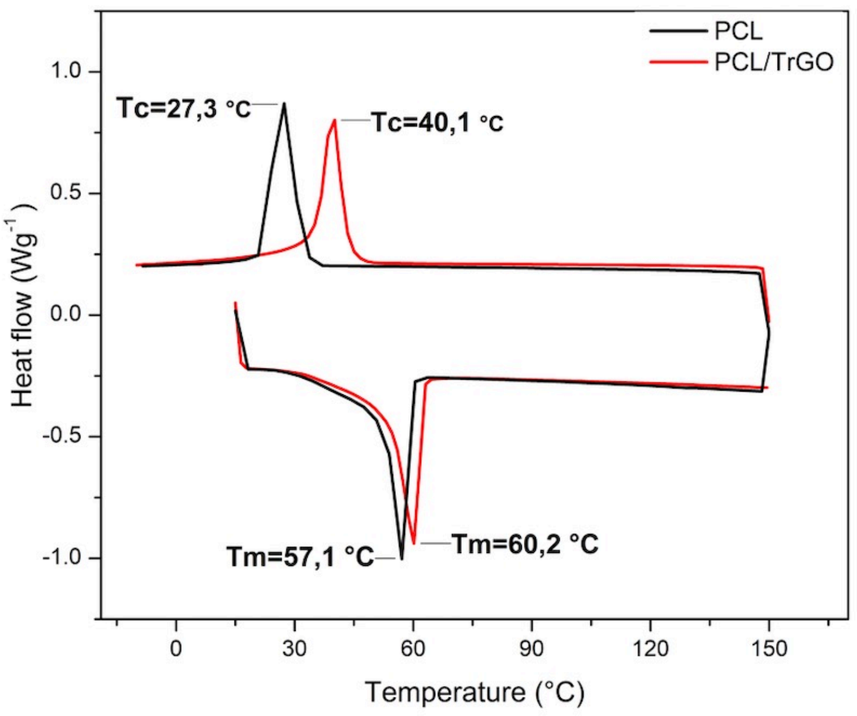

Figure S2: DSC curve of PCL and PCL/TrGO.

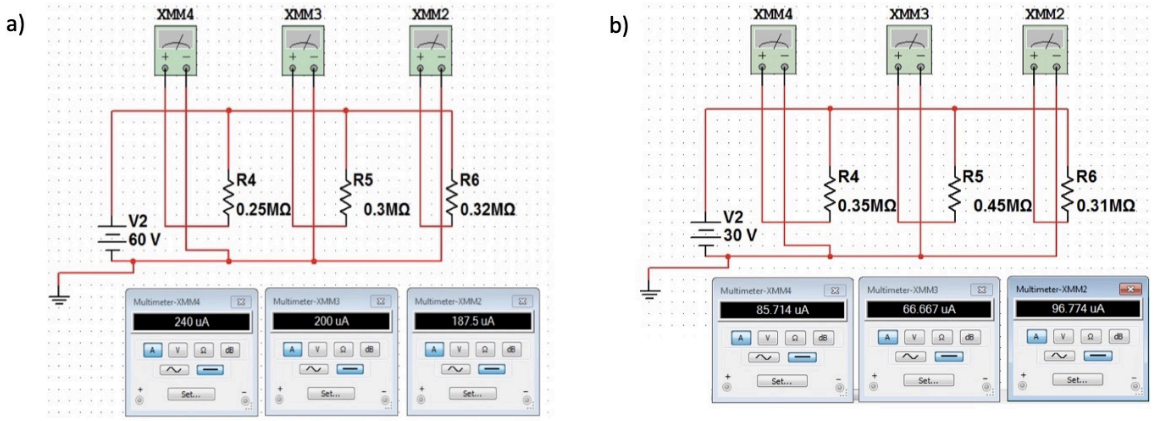

Figure S3: Simulation of the experimental set-up using NI Multisim^{TM}, using a) 60 V and b) 30 V for the estimation of the current value in each scaffolds.

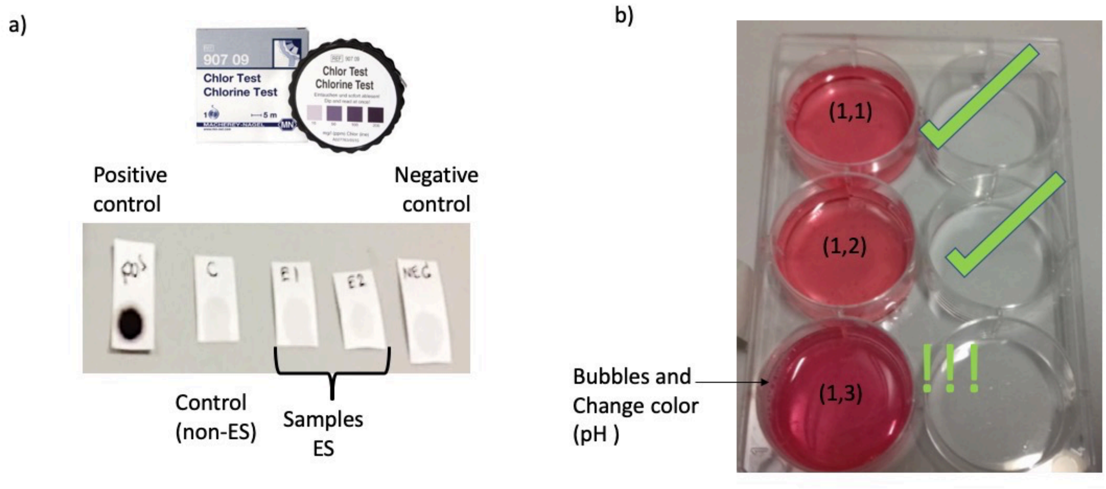

Figure S4: a) Chlorine test paper (Macherey-nagel) in contact with the samples after ES showing that there was no Chlorine species generated. Positive control samples were 1% w/v Rely+On™ Virkon® (DuPont) and negative comtrol samples were deionized water. b) Color change and gas production in the DMEM culture medium (1,3) caused by poor electrical insulation of the carbon electrodes with silicone rubber causing current leakage into the culture medium.
